# Inhibition of the RAGE products increases survival in experimental models of severe sepsis and systemic infection

**DOI:** 10.1186/cc6184

**Published:** 2007-11-06

**Authors:** Emily C Lutterloh, Steven M Opal, Debra D Pittman, James C Keith, Xiang-Yang Tan, Brian M Clancy, Helen Palmer, Kim Milarski, Ying Sun, John E Palardy, Nicholas A Parejo, Noubar Kessimian

**Affiliations:** 1 Division of Infectious Diseases, Memorial Hospital of Rhode Island, 111 Brewster St., Pawtucket, RI 02860; 2 Wyeth Research, 35 Cambridge Park Dr., Cambridge, MA 02140; 3 Department of Pathology, Memorial Hospital of Rhode Island, 111 Brewster St., Pawtucket, RI 02860

## Abstract

**Introduction:**

The receptor for advanced glycation end products (RAGE), a multi-ligand member of the immunoglobulin superfamily, contributes to acute and chronic disease processes, including sepsis.

**Methods:**

We studied the possible therapeutic role of RAGE inhibition in the cecal ligation and puncture (CLP) model of polymicrobial sepsis and a model of systemic listeriosis using mice genetically deficient in RAGE expression or mice injected with a rat anti-murine RAGE monoclonal antibody.

**Results:**

The 7-day survival rates after CLP were 80% for RAGE^-/- ^mice (*n *= 15) (*P *< 0.01 versus wild-type), 69% for RAGE^+/- ^mice (*n *= 23), and 37% for wild-type mice (*n *= 27). Survival benefits were evident in BALB/c mice given anti-RAGE antibody (*n *= 15 per group) over serum-treated control animals (*P *< 0.05). Moreover, delayed treatment with anti-RAGE antibody up to 24 hours after CLP resulted in a significant survival benefit compared with control mice. There was no significant increase in tissue colony counts from enteric Gram-negative or Gram-positive bacteria in animals treated with anti-RAGE antibody. RAGE^-/-^, RAGE^+/-^, and anti-RAGE antibody-treated animals were resistant to lethality from *Listeria monocytogenes *by almost two orders of magnitude compared with wild-type mice.

**Conclusion:**

Further studies are warranted to determine the clinical utility of anti-RAGE antibody as a novel treatment for sepsis.

## Introduction

Sepsis is a major clinical problem in acute care medicine and surgery, and treatment options remain limited [1,2]. This unmet medical need has inspired a great deal of work to understand the molecular pathogenesis of sepsis and to develop improved therapeutic interventions. One molecule that has been implicated in the pathogenesis of sepsis is the receptor for advanced glycation end products (RAGE), a member of the immunoglobulin (Ig) superfamily [3,4]. It consists of an extracellular domain comprised of an Ig-like V-type domain and two Ig-like C-type domains, a single membrane-spanning domain, and a cytosolic tail [3]. The V-type domain and the cytoplasmic domain are important for ligand binding and for intracellular signaling, respectively. In addition to membrane-bound RAGE, soluble forms of RAGE (sRAGE) have been detected in plasma. Although the physiologic function of RAGE is unclear, it is involved in the inflammatory response and may have a role in neural development [5].

In several animals models, modulation of RAGE expression or activity has reduced inflammatory responses. In a model of delayed-type hypersensitivity, mice sensitized to methylated bovine serum albumin (mBSA) and administered sRAGE or anti-RAGE antibody (F(ab)_2 _fragment) had decreased inflammation following mBSA challenge [6]. In a study of chronic inflammation using an interleukin (IL)-10 null model of colitis, 6 weeks of treatment with sRAGE decreased the number of mice with colitis [6]. In streptozotocin-treated diabetic mice, sRAGE reduced periodontitis in mice challenged with *Porphyromonas gingivalis *[7]. Additionally, sRAGE reduced neutrophil extravasation into the peritoneum in thioglycollate-induced peritonitis in diabetic mice [8]. Reduced neutrophil migration into the peritoneum was also observed in RAGE^-/- ^mice [9]. These studies suggest a role for RAGE in several disease settings.

RAGE is expressed at low levels on multiple cell types. Expression is increased upon ligand interaction in chronic disease states such as rheumatoid arthritis [8,10] and diabetic nephropathy [11]. Ligands include advanced glycation end products (AGEs) which form in prolonged hyperglycemic states. However, AGEs may be only incidental, pathogenic ligands [6,12]. RAGE is a pattern-recognition receptor that binds diverse classes of endogenous molecules. Known ligands include high-mobility group box-1 (HMGB-1) [12], the S100/calgranulins [6], and peptides with a three-dimensional structure consisting of beta-sheet fibrils, such as amyloid [5,13]. RAGE is also a counter-receptor for the beta_2_-integrins Mac-1 and p150, 95 [9]. RAGE is part of a newly appreciated component of the innate immune system referred to as the damage-associated molecular pattern system [14,15].

HMGB-1 is an inflammatory cytokine and RAGE ligand that may be important in the septic response [6,12,16]. HMGB-1, also a DNA-binding protein, is released from cells due to necrosis or via a non-classical secretion pathway and is a late-stage mediator of lethality in a murine model of sepsis. Many of the RAGE ligands represent a unique class of molecules with both intra- and extracellular activities [14,15]. In a study using the cecal ligation and puncture (CLP) model of polymicrobial sepsis, HMGB-1 levels increased over the span of 1 to 2 days after CLP and remained elevated during the course of disease [17]. Mortality decreased in this model with administration of anti-HMGB-1 antibody. An *in vitro *study showed that activation of human umbilical venular endothelial cells by HMGB-1 was partially decreased by anti-RAGE antibodies given with the HMGB-1 [18]. The anti-RAGE antibodies did not affect stimulation with lipopolysaccharide or tumor necrosis factor (TNF)-α, implying that some elements of the inflammatory response induced by HMGB1 are mediated by RAGE.

In a study by Liliensiek and colleagues [19], the genomic deletion of RAGE resulted in a decreased septic response, 80% of RAGE knockout mice survived the lethal effects of CLP, whereas only 20% of wild-type mice survived. Administration of sRAGE to block RAGE ligands also increased survival to 40% compared with 17% in the control group. There were fewer inflammatory cells on the peritoneum of RAGE^-/- ^mice compared with wild-type mice after CLP; however, cytokine levels and inflammatory cells in the blood were similar between wild-type mice and RAGE^-/- ^mice. The study suggests that RAGE may be a mediator in polymicrobial sepsis and that modulation may affect the pathophysiology.

We hypothesized that treatment with an anti-RAGE antibody may have a protective effect in polymicrobial sepsis. Our experiments show that the administration of a rat anti-murine RAGE increased survival in the CLP model of sepsis compared with mice given control serum, even with delayed administration as long as 24 hours after CLP. Additionally, our studies confirm the finding that homozygous RAGE knockout mice survive polymicrobial sepsis at a higher rate compared with wild-type mice and show that heterozygous RAGE^+/- ^mice have a similar survival rate. Finally, we show that RAGE^-/- ^and RAGE^+/- ^mice and RAGE monoclonal antibody (mAb)-treated mice also survive a systemic *Listeria monocytogenes *challenge significantly better than wild-type mice. These results demonstrate that RAGE may play an important role in the pathogenesis of sepsis in the CLP model and that anti-RAGE mAbs can increase the survival of septic mice.

## Materials and methods

### Materials and bacterial strains

Reagents and chemicals were purchased from Sigma-Aldrich (St. Louis, MO, USA) unless otherwise stated. The IgG2b anti-murine RAGE mAb was developed by Wyeth (Cambridge, MA, USA). This mAb is a high-affinity rat-derived anti-mouse RAGE antibody with a binding affinity of 0.3 nM for murine dimeric RAGE and binds to the N-terminal region of RAGE. Specificity of the antibody was demonstrated by binding to RAGE on the cell surface, direct binding to sRAGE, and immunohistochemistry (manuscript in preparation). Endotoxin levels were measured in all antibody preparations and were less than 1 endotoxin unit/mg protein. The high-affinity IgG hamster anti-TNF-α neutralizing mAb TN3.1912 was provided as a gift from Celltech LTD (Slough, Berkshire, UK). The challenge strain of *L. monocytogenes *was purchased from American Type Culture Collection (ATCC # 19115; Manassas, VA, USA).

### Animal strains and animal husbandry

Mouse strains used in these experiments were 2 to 6 months old and were specific-pathogen-free. BALB/c mice (Charles River Laboratories, Inc., Wilmington, MA, USA), homozygous RAGE^-/- ^129SvEvBrd male mice, heterozygous RAGE^+/- ^129SvEvBrd male mice, and wild-type RAGE^+/+ ^129SvEvBrd male mice (bred in-house at Wyeth) were used. The RAGE knockout mouse was designed at Wyeth Research (generated at Lexicon Genetics Incorporated, The Woodlands, TX, USA) as a gene targeted conditional knockout in 129SvEv-Brd mice in which Cre recombinase excises exons 2, 3, and 4. The resulting deletion results in a frame shift and truncation of the RAGE protein, and protein is not produced. The absence of RAGE protein in the RAGE^-/- ^mice was confirmed by Western immunoblot analysis of lung tissue lysates. Lungs were harvested from individual RAGE^+/+^, RAGE^+/-^, and RAGE^-/- ^animals and were flash-frozen. The tissue was lysed with radio-immunoprecipitation assay buffer. Equivalent amounts of total protein from each sample were analyzed by electrophoresis on a NuPAGE 4% to 12% Bis-Tris gel (Invitrogen Corporation, Carlsbad, CA, USA) and detected with immunodetection with the RAGE mAb or actin antibody and a horseradish peroxidase-conjugated anti-donkey antibody using standard protocols. RAGE is not essential for viability in mice; RAGE null mice have no obvious phenotype and breed normally.

Animals were housed in an Institutional Animal Care and Use Committee (IUCAC)-approved facility under Biosafety Level 2 conditions and monitored by Brown University veterinary staff. Cages were covered with high-efficiency particulate air filters and maintained at constant ambient temperature and humidity with 12-hour day-night cycling. Animals were provided with an *ad libitum *supply of commercial mouse chow and distilled water and allowed to adjust to laboratory conditions for at least 7 days before undergoing experimental procedures. The experimental protocol was approved by the IUCAC before any procedures were undertaken.

### Cecal ligation and puncture model

The CLP procedure has been described previously [20]. Briefly, animals were anesthetized with an intraperitoneal injection of 200 μL of a combination of ketamine (Bedford Laboratories, Bedford, OH, USA) (9 mg/mL) and xylazine (Phoenix Pharmaceutical, Inc., St. Joseph, MO, USA) (1 mg/mL). The cecum was exteriorized through a midline abdominal incision of approximately 1 cm. The cecum was ligated with 5.0 monofilament just distal to the ileocecal junction (greater than 90% of the cecum ligated). The ante-mesenteric side of the cecum was punctured through and through with a 23-gauge needle. A scant amount of luminal content was expressed through both puncture sites to ensure patency. The cecum was returned to the abdominal cavity, and the fascia and skin incisions were closed with 6.0 monofilament and surgical staples, respectively. Topical 1% lidocaine and bacitracin were applied to the surgical site post-operatively. All animals received a single intramuscular injection of trovafloxacin (Pfizer Inc, New York, NY, USA) at a dose of 20 mg/kg and subcutaneous fluid resuscitation with 1.0 mL of normal saline immediately post-operatively. Animals were then returned to their individual cages and rewarmed using heat lamps until they regained normal posture and mobility.

Anti-RAGE mAb at 7.5 mg/kg or 15 mg/kg (or 1% autologous mouse serum control) was given once intravenously to wild-type 129SvEvBrd or BALB/c mice 30 to 60 minutes before CLP or at the following time intervals after CLP: 6, 12, 24, or 36 hours. Five animals underwent sham surgery (laparotomy with exteriorization of the cecum but without ligation or puncture). Mice were assessed for survival up to 7 days following surgery.

### Murine listeriosis challenge model

A standard inoculum of *L. monocytogenes *was prepared from cultures grown 18 hours at 37°C in trypticase soy broth (TSB) (BBL, Cockeysville, MD, USA). Bacteria were centrifuged at 10,000*g *for 15 minutes at 4°C and resuspended in phosphate-buffered saline. Bacterial concentrations were adjusted spectrophotometrically and checked by quantitative dilutional plate counts on trypticase soy agar plates with 5% sheep red blood cells (BBL). Serial dilutions ranging from 10^3 ^to 10^6 ^colony-forming units (CFU) of *L. monocytogenes *were administered intravenously to determine the median lethal dose (LD_50_) for wild-type mice, homozygous RAGE^-/- ^knockouts, RAGE^+/- ^heterozygotes, and BALB/c mice given 15 mg/kg anti-RAGE mAb intravenously 1 hour before bacterial challenge. Animals were followed for 7 days after the administration of the intravenous challenge with *L. monocytogenes*. Survivors were euthanized for tissue analysis and microbiologic study.

For the detailed differential quantitative microbiology and cytokine determinations, a standard inoculum of 10^4 ^CFU was given intraperitoneally 1 hour after an intravenous infusion of anti-RAGE mAb (15 mg/kg), anti-TNF mAb (20 mg/kg), or equal volume of 1% autologous murine serum as a control. RAGE^+/+^, RAGE^+/-^, and RAGE^-/- ^mice were also studied (*n *= 5 per group). Animals were euthanized 48 hours after *L. monocytogenes *challenge, and quantitative microbiology was performed from liver and spleen tissues by mincing the tissue samples with serial dilution on blood agar plates.

Injections and manipulations of the animals were conducted under light CO_2 _anesthesia to minimize stress to the experimental animals. Animals were monitored daily for survival. Moribund animals (hypothermia below 33°C and inactive with loss of righting response) were euthanized and scored as lethally infected animals.

### Tissue collection and cytokine measurement

Quantitative microbiology was performed from organ samples obtained at necropsy in both the CLP and listeriosis experiments. Blood samples were obtained from surviving animals at the time of sacrifice, and serum was collected and immediately placed on ice for cytokine determination. Serum cytokines were measured by an enzyme-linked immunosorbent multiplex assay using the custom-made plates and protocol provided by Meso Scale Discovery (Gaithersburg, MD, USA). Cytokines assayed were monocyte chemoattractant protein-1 (MCP-1), IL-1-β, TNF-α, interferon-γ, and IL-6. Tissue samples were collected from the lung, liver, and spleen. Peritoneal fluid was obtained by lavaging the peritoneal cavity with 5 mL of sterile saline and withdrawing the fluid. Organ tissues were weighed and then pulverized to generate a suspension of tissue in TSB. Specimens were serially diluted and cultured at 37°C aerobically on TSB (for Gram-negative and Gram-positive bacteria) and MacConkey agar (for Gram-negative bacteria) to obtain quantitative bacterial counts standardized per gram of organ weight or CFU per milliliter of peritoneal lavage fluid.

Tissues (lung, distal ileum) were analyzed by a pathologist blinded to the treatment assignment of each animal and scored on a defined pathology score graded from 0 (normal) to 4 (diffuse and extensive necrosis). Total lung water as a measure of pulmonary edema was calculated from wet-to-dry ratios of lung tissue.

### Statistical design and data analysis

The primary endpoint in each experiment was survival. Experiments were performed using a numeric code that blinded the investigators to the animal genotype or antibody treatment (versus serum control) until completion of the study. Numeric data are presented as mean (± standard error of the mean). Differences in survival were analyzed by a Kaplan-Meier survival plot and the log-rank statistic. The non-parametric one-way analysis of variance statistic Kruskal-Wallis test (for multiple groups) or the Mann-Whitney *U *test (for two groups) was used to analyze differences between groups. The Dunn multiple comparisons post-test was used to confirm differences when analyzing comparisons involving multiple groups. A two-tailed *P *value of less than 0.05 was considered significant.

## Results

### Survival of homozygous RAGE knockouts, RAGE heterozygotes, and wild-type animals after cecal ligation and puncture

**Figure 1:**
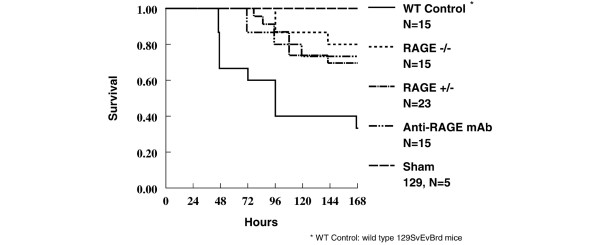
Modulation of receptor for advanced glycation end products (RAGE) protects mice from the effects of cecal ligation and puncture (CLP). Kaplan-Meier survival analysis following CLP comparing wild-type RAGE^+/+ ^129SvEvBrd mice (*n *= 15), RAGE^-/- ^mice (*n *= 15), RAGE^+/- ^mice (*n *= 23), and anti-RAGE monoclonal antibody-treated (15 mg/kg intravenously 30 to 60 minutes before CLP) wild-type mice (*n *= 15). *P *< 0.001 for each group in comparison with the wild-type CLP control group. Sham surgery-treated wild-type 129SvEvBrd mice (*n *= 5) were used as an additional control group. WT, wild-type.

Homozygous RAGE knockouts (*n *= 15) and RAGE heterozygotes (*n *= 23) showed a significant degree of protection from the lethal effects of CLP compared with wild-type RAGE ^+/+ ^animals (*n *= 15) (*P *< 0.001) (Figure 1). RAGE heterozygotes were protected from lethal polymicrobial sepsis nearly as well as the homozygous RAGE knockouts (RAGE^-/- ^versus RAGE^+/-^; *P *= not significant [ns]). As expected, sham surgery animals (*n *= 5) all survived. An additional group of 15 wild-type 129SvEvBrd animals were given anti-RAGE mAb 30 to 60 minutes before CLP and had a survival advantage similar to that of the RAGE knockouts when compared with the wild-type, serum-treated, control animals (Figure 1).

**Figure 2:**
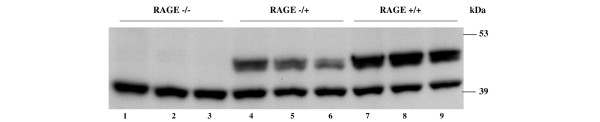
Receptor for advanced glycation end products (RAGE) protein expression in lung tissue from individual RAGE^-/- ^(lanes 1 to 3), RAGE^+/- ^(lanes 4 to 6), and RAGE^+/+ ^(lanes 7 to 9) animals. Actin was used to demonstrate equal loading.

The expression of RAGE protein was examined by Western immunoblot analysis of lung tissue where RAGE is constitutively expressed at high levels. In contrast to the expression of RAGE in RAGE ^+/+ ^mice, no RAGE protein was detected in the lungs of RAGE^-/- ^mice. The levels of expression in RAGE^+/- ^mice were reduced compared with wild-type mice (Figure 2).

Tissue colony counts for aerobic Gram-positive and Gram-negative enteric bacterial organisms following CLP were assessed to determine whether RAGE knockouts, RAGE heterozygotes, or anti-RAGE mAb-treated mice were different from wild-type mice or control-treated mice. No significant differences were found in liver and splenic tissues and peritoneal fluid, except that all were significantly higher than in sham-operated animals. The homozygous RAGE knockouts had the lowest amount of lung water compared with other groups, although this did not reach significance (wet-to-dry ratio: 4.8 ± 0.2 RAGE^-/- ^versus 5.0 ± 0.4 RAGE^+/- ^versus 5.3 ± 0.3 wild-type; *P *= ns).

### Effects of anti-RAGE antibody with cecal ligation and puncture

**Figure 3:**
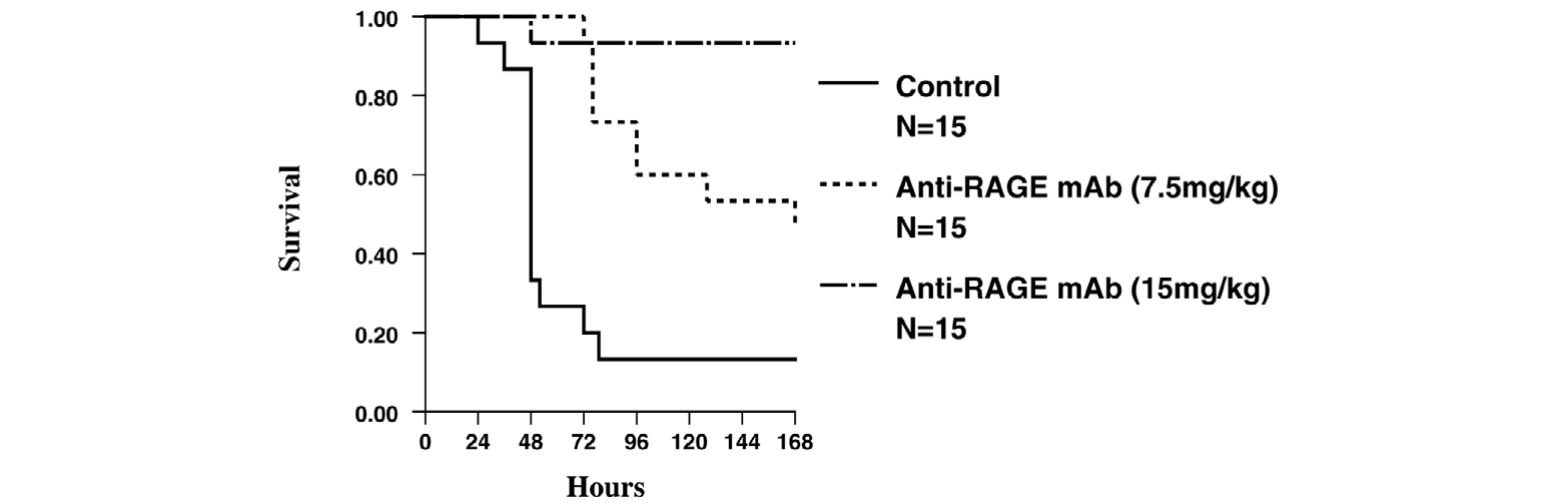
Lethality from cecal ligation and puncture (CLP) is decreased in BALB/c mice administered an anti-RAGE monoclonal antibody (mAb). The Kaplan-Meier survival analysis following CLP compares anti-RAGE mAb-treated animals given 7.5 mg/kg (*n *= 15) or 15 mg/kg (*n *= 15) intravenously 30 to 60 minutes before CLP with serum control animals (*n *= 15). The group given 15 mg/kg had a significantly greater survival than the group given 7.5 mg/kg (*P *< 0.05) or serum control (*P *< 0.001). RAGE, receptor for advanced glycation end products.

There was a significant difference in survival in BALB/c animals given control serum (*n *= 15) compared with animals given anti-RAGE antibody (7.5 mg/kg group [*n *= 15] or 15 mg/kg group [*n *= 15]) 30 to 60 minutes before CLP (Figure 3). Optimal protective effects were achieved at 15 mg/kg of anti-RAGE mAb (*P *< 0.05 versus 7.5 mg/kg group; *P *< 0.001 versus serum control), and this dose was employed in subsequent experiments with delayed mAb treatment. Animals given anti-RAGE antibody did not have significantly increased organ bacterial loads compared with control animals, but both groups had significantly more CFU/g of spleen and liver tissue than sham-treated control animals (Table 1). Histopathology of lung tissue and small bowel mucosa at necropsy was markedly abnormal in the serum control group, whereas pathological findings were significantly reduced in the anti-RAGE mAb group and the sham surgery group (Table 1).

**Table 1:** Microbiologic and pathologic findings following anti-RAGE mAb therapy in cecal ligation and puncture

Parameter	Sham	Serum control	Anti-RAGE mAb (15 mg/kg)
Number of mice	5	15	15
Aerobic Gram-negative bacteria (CFU/g)	0.6 ± 1.5^a^	5,643 ± 1,281	4,910 ± 395
Aerobic Gram-positive bacteria (CFU/g)	601 ± 548^a^	15,616 ± 6,800	11,222 ± 1,873
Pathology score (lung, small bowel)	0.6 ± 0.5	3.0 ± 0.9^b^	1.8 ± 1.1
Wet-to-dry ratio (lung tissue)	4.6 ± 0.6	5.3 ± 0.5	5.1 ± 0.6

^a^*P *< 0.05 sham versus other groups; ^b^*P *< 0.005 control versus sham or anti-RAGE mAb. CFU, colony-forming units; mAb, monoclonal antibody; RAGE, receptor for advanced glycation end products.

### Survival of animals with delayed administration of anti-RAGE mAb after cecal ligation and puncture

**Figure 4:**
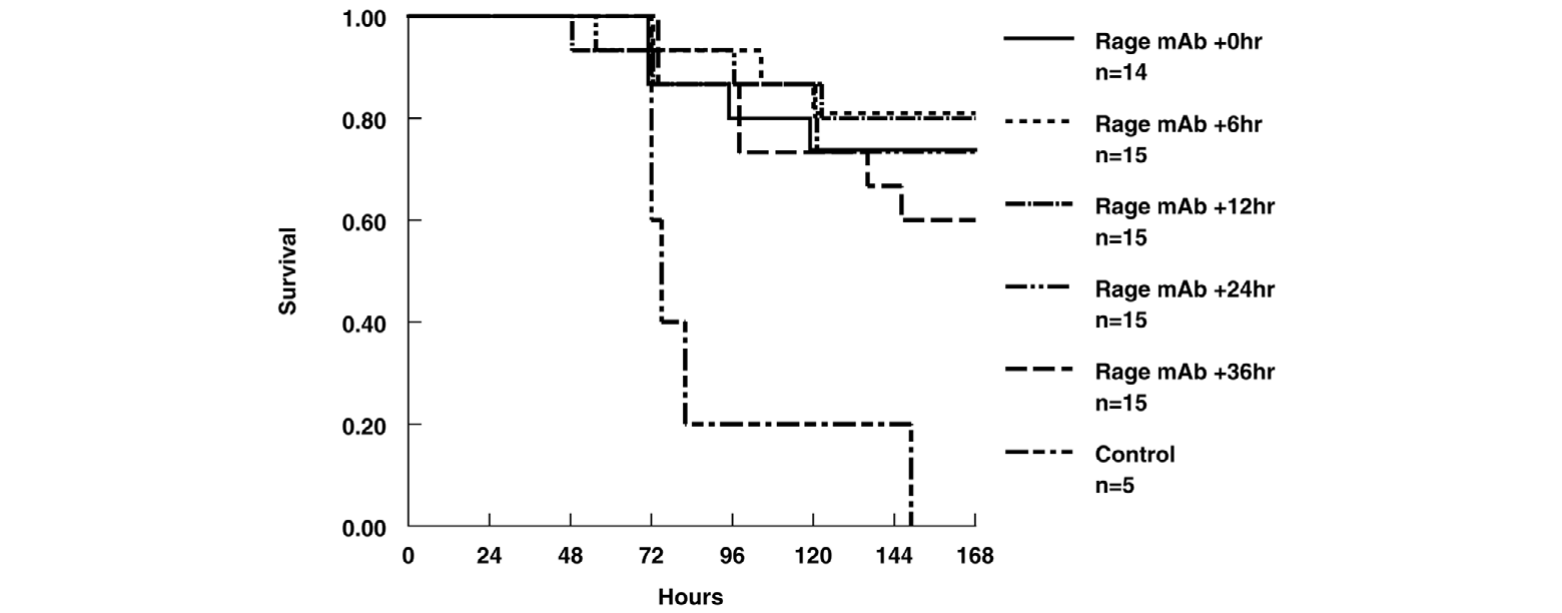
Delayed administration of the anti-RAGE antibody is protective in cecal ligation and puncture (CLP). The Kaplan-Meier survival analysis following CLP in BALB/c mice compares delayed anti-RAGE monoclonal antibody (mAb) treatment given at various time intervals after CLP with serum control. Each group had a significantly greater survival than the control group (*P *< 0.01), except for the 36-hour delayed treatment group (*P *= 0.12). RAGE, receptor for advanced glycation end products.

The effects of delayed administration of a single 15 mg/kg dose of anti-RAGE antibody are shown in Figure 4. The administration of RAGE mAb provided significant protection up to 24 hours after CLP (*P *< 0.01). Delayed mAb administration 36 hours after CLP showed a favorable survival trend, but the differences were no longer significant compared with the serum-treated control group (*P *= 0.12). The tissue concentrations of aerobic enteric Gram-negative and Gram-positive bacteria did not differ between treatment groups (*P *= ns).

### *Listeria monocytogenes *challenge

When challenged with *L. monocytogenes*, the LD_50 _(log_10_) values were 3.30 ± 0.12 for BALB/c mice, 3.31 ± 0.2 CFU for RAGE^+/+ ^129SvEvBrd mice, 5.98 ± 0.39 for RAGE^+/- ^mice, and 5.10 ± 0.47 for RAGE^-/- ^mice. This difference of more than two orders of magnitude in LD_50 _from systemic listeriosis was statistically significant (*P *< 0.01) for both the RAGE heterozygotes and homozygotes compared with wild-type mice. A single dose of anti-RAGE antibody also provided BALB/c mice significant protection from lethal systemic listeriosis with an LD_50 _of 4.69 ± 0.55 (*P *< 0.05 versus BALB/c control). The level of protection against listeriosis provided by the anti-RAGE mAb was similar to that observed in RAGE^-/- ^animals but was not as great as that afforded RAGE^+/- ^animals (*P *< 0.05).

**Figure 5:**
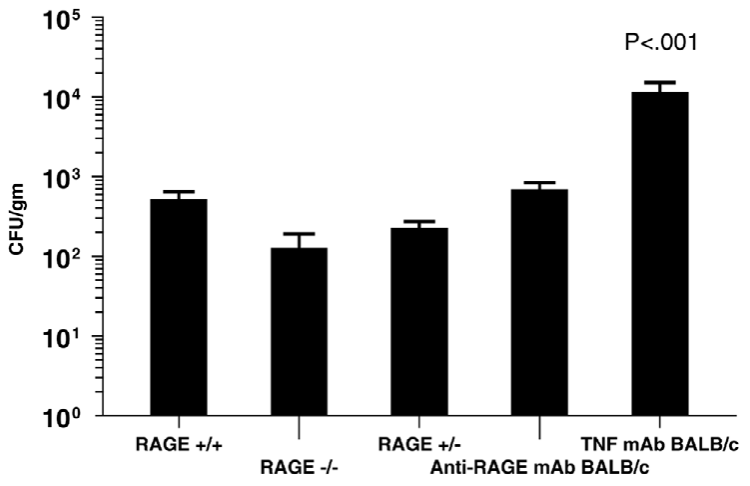
Inhibition or deletion of receptor for advanced glycation end products (RAGE) does not disrupt the host mechanism for clearance of *Listeria monocytogenes*. Quantitative levels of *L. monocytogenes *in organ samples (liver and spleen, *n *= 10 per group) 48 hours after an intraperitoneal injection of 10^4 ^colony-forming units (CFU) per animal (*P *< 0.001 anti-tumor necrosis factor [TNF] monoclonal antibody [mAb] group versus all other groups).

There was no statistically significant difference in the quantity of *L. monocytogenes *isolated in liver and spleen tissues following a standard systemic challenge of 10^4 ^CFU among groups (*n *= 10 per group) of RAGE^+/+ ^mice, RAGE^-/- ^mice, RAGE^+/- ^mice, or BALB/c mice given anti-RAGE mAb (Figure 5). However, as expected, there was a highly statistically significant increase in organ bacterial concentrations in BALB/c mice given the same inoculum of *L. monocytogenes *following the administration of an anti-TNF antibody (*P *< 0.001).

**Figure 6:**
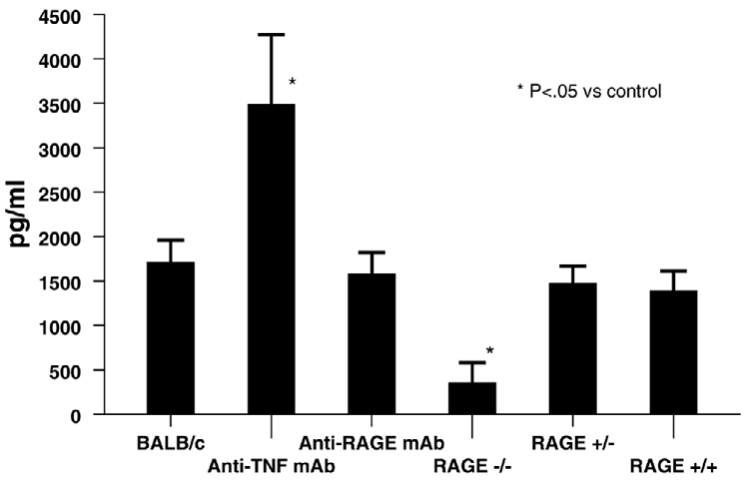
Interferon-gamma levels following intraperitoneal challenge with 10^4 ^colony-forming units (CFU) of *Listeria monocytogenes *are shown (*n *= 10 for each group). *P *< 0.05 for anti-tumor necrosis factor (TNF) monoclonal antibody (mAb) group and RAGE^-/- ^group, both versus control group. RAGE, receptor for advanced glycation end products.

Cytokine determinations after *Listeria *challenge showed a significantly lower level of interferon-γ in RAGE^-/- ^mice compared with RAGE^+/+ ^mice (Figure 6). The BALB/c mice administered anti-TNF mAb had a significantly higher level of interferon-γ compared with BALB/c controls, whereas the animals given anti-RAGE mAb had interferon-γ levels that were not statistically different compared with control animals. Similar results were observed with IL-6 (anti-TNF mAb group 459 ± 121 pg/mL versus control group 38 ± 14 pg/mL; *P *< 0.01) and MCP-1 (anti-TNF mAb 1,363 ± 480 pg/mL versus control group 566 ± 70 pg/mL; *P *< 0.05). No significant differences were found in IL-6 or MCP-1 levels in RAGE-deficient animals or in anti-RAGE antibody-treated mice compared with the control group. Other cytokine determinations showed no significant differences.

## Discussion

These results support the beneficial effects of treatment with anti-RAGE antibody in a standard murine model of polymicrobial, intra-abdominal sepsis. We have confirmed that homozygous RAGE knockouts have increased survival compared with wild-type mice in the CLP model of sepsis. In addition, heterozygous RAGE^+/- ^mice were similarly protected compared with wild-type mice. RAGE suppression did not increase tissue colony counts from enteric Gram-negative or Gram-positive bacteria. These results show that the modulation of RAGE protected mice from the lethal effects of CLP-induced sepsis. RAGE expression is highly detrimental to animals challenged systemically with *L. monocytogenes *as evidenced by the marked survival benefits observed in homozygous RAGE knockouts and heterozygotes compared with wild-type animals. The precise mechanism of these benefits is unclear and under investigation.

The finding that homozygous and heterozygous RAGE knockout mice survive sepsis at higher rates than wild-type animals seems counterintuitive from an evolutionary standpoint. Speculation as to the reason for the persistence of a widely expressed and seemingly disadvantageous gene in the mammalian genome is hampered by the lack of a detailed understanding of RAGE's physiologic role. An intriguing finding is that, contrary to many systems designed to maintain homeostasis, RAGE expression is increased in inflammatory states when it binds to its ligands [21,22]. This positive feedback mechanism might function counter to homeostatic mechanisms in the presence of a massive inflammatory insult and hasten death of a severely ill animal.

Given the finding of increased survival with polymicrobial sepsis in RAGE knockout animals, we hypothesized that treatment with an anti-RAGE antibody would increase survival. Our RAGE knockout animals, however, may have developed compensatory mechanisms that alter their inflammatory response. The experiment showing increased survival in animals administered the anti-RAGE antibody confirms that the benefit is present in situations in which physiologic compensation for genetic deficiency could not have occurred. This opens up an entirely new avenue for the treatment of severe sepsis and suggests that inhibition of the RAGE pathway may be an effective approach for treatment of sepsis in clinical settings.

Activation of the RAGE receptor triggers signaling cascades leading to sustained cellular activation, increase of RAGE itself, and propagation of the response. HMGB-1 and other RAGE ligands may contribute to damage and host response as part of the danger-associated molecular pattern system [14,15]. In animal models, HMGB-1 is a mediator of severe sepsis, and an anti-HMGB-1 antibody decreased mortality in the CLP model of sepsis [6,12,16]. In this study, the administration of anti-RAGE mAb and a genetic deletion of RAGE conferred resistance to the lethal effects of CLP. Additionally, delayed treatment with the anti-RAGE antibody up to 24 hours after CLP resulted in a significant survival benefit, suggesting that disruption of the interaction between RAGE and RAGE ligands is beneficial in this setting.

Systemic *L. monocytogenes *challenge is a classic model for study of the innate and acquired immune response in mice [23,24]. RAGE blockade or insufficiency due to administration of anti-RAGE antibody or as observed in RAGE null or RAGE heterozygous animals did not produce significant sensitivity to the *Listeria *challenge compared with wild-type animals, indicating that the deleterious effects of RAGE are present in an inflammatory state other than that accompanying polymicrobial sepsis. Mice administered anti-RAGE mAb and RAGE knockout animals appear to clear *L. monocytogenes *as well as wild-type animals. This is in contrast to animals given anti-TNF antibody in which the *L. monocytogenes *colony counts in tissue samples were markedly increased. Previous studies have demonstrated that anti-TNF antibodies suppress the innate immune response and increase the sensitivity to challenge. Similarly, cytokine levels were increased after *Listeria *challenge in animals given anti-TNF mAb. However, the levels were similar in animals given the anti-RAGE antibody and the control animals, consistent with no increase in sensitivity to the *Listeria *challenge.

The finding of a survival benefit after delayed administration of anti-RAGE antibody was encouraging. This may have important clinical implications since an intervention such as anti-RAGE antibody treatment typically cannot be given immediately after the inciting event in septic patients.

## Conclusion

These data provide support for the use of anti-RAGE mAb as a salvage therapy for patients with established severe sepsis. Inhibition of the RAGE pathway may be an effective approach for treatment of sepsis in clinical settings. Further pre-clinical and clinical investigations will be necessary to determine the feasibility of this strategy in humans with life-threatening sepsis or septic shock.

## Key messages

• Receptor for advanced glycation end products (RAGE) knockout mice survive sepsis associated with cecal ligation and puncture better than do wild-type mice.

• Administration of anti-RAGE monoclonal antibody improves survival in a murine model of polymicrobial sepsis, even when delayed up to 24 hours.

• Administration of anti-RAGE monoclonal antibody is protective in a murine model of systemic listeriosis.

## Abbreviations

AGE = advanced glycation end products; CFU = colony-forming units; CLP = cecal ligation and puncture; HMGB-1 = high-mobility group box-1; Ig = immunoglobulin; IL = interleukin; IUCAC = Institutional Animal Care and Use Committee; LD_50 _= median lethal dose; mAb = monoclonal antibody; mBSA = methylated bovine serum albumin; MCP-1 = monocyte chemoattractant protein-1; ns = not significant; RAGE = receptor for advanced glycation end products; sRAGE = soluble receptor for advanced glycation end products; TNF = tumor necrosis factor; TSB = trypticase soy broth.

## Competing interests

ECL has received grant support from Wyeth Research. SMO has received grant support from Wyeth Research and clinical coordinating center support from Novartis International AG in Switzerland and Eisai Inc. (Woodcliff Lake, NJ, USA). DDP, JCK, X-YT, BMC, HP, KM, and YS are employees of Wyeth Research and own company stock. The other authors declare that they have no competing interests.

## Authors' contributions

ECL participated in study design and acquisition and interpretation of data and drafted the manuscript. SMO participated in study design, acquisition of data, and interpretation of data and helped draft the manuscript. DDP participated in conception of the study, analysis of RAGE knockouts and antibody, and revision of the manuscript. JCK participated in study design and interpretation of data. X-YT participated in the generation and characterization of RAGE antibody. BMC, HP, YS, and NAP participated in acquisition of data. KM participated in generation of RAGE knockouts. JEP participated in acquisition of data and performed statistical analysis. NK performed analysis of pathologic specimens. All authors read and approved the final manuscript.
